# Organization and distribution of glomeruli in the bowhead whale olfactory bulb

**DOI:** 10.7717/peerj.897

**Published:** 2015-04-28

**Authors:** Takushi Kishida, JGM Thewissen, Sharon Usip, Robert S. Suydam, John C. George

**Affiliations:** 1 Wildlife Research Center, Kyoto University, Kyoto, Japan; 2 Department of Anatomy and Neurobiology, Northeast Ohio Medical University, Rootstown, OH, USA; 3 Department of Wildlife Management, North Slope Borough, Barrow, AK, USA

**Keywords:** Brain, Baleen whale, Cetacea, Olfactory marker protein, Mysticeti, Olfactory receptor

## Abstract

Although modern baleen whales (Mysticeti) retain a functional olfactory system that includes olfactory bulbs, cranial nerve I and olfactory receptor genes, their olfactory capabilities have been reduced to a great degree. This reduction likely occurred as a selective response to their fully aquatic lifestyle. The glomeruli that occur in the olfactory bulb can be divided into two non-overlapping domains, a dorsal domain and a ventral domain. Recent molecular studies revealed that all modern whales have lost olfactory receptor genes and marker genes that are specific to the dorsal domain. Here we show that olfactory bulbs of bowhead whales (*Balaena mysticetus*) lack glomeruli on the dorsal side, consistent with the molecular data. In addition, we estimate that there are more than 4,000 glomeruli elsewhere in the bowhead whale olfactory bulb, which is surprising given that bowhead whales possess only 80 intact olfactory receptor genes. Olfactory sensory neurons that express the same olfactory receptors in rodents generally project to two specific glomeruli in an olfactory bulb, implying an approximate 1:2 ratio of the number of olfactory receptors to the number of glomeruli. Here we show that this ratio does not apply to bowhead whales, reiterating the conceptual limits of using rodents as model organisms for understanding the initial coding of odor information among mammals.

## Introduction

Terrestrial mammals generally have a well-developed sense of smell that can discriminate millions of odors using hundreds or thousands of olfactory receptors (ORs) (Nei, Niimura & Nozawa, 2008). Odorants are detected by ORs expressed in the cell membrance of the olfactory sensory neurons (OSNs), which project to the glomeruli of the olfactory bulbs (OBs). Each OSN expresses only one *OR* gene (Serizawa, Miyamichi & Sakano, 2004), and OSNs expressing the same OR converge their axons to a specific set of glomeruli in the olfactory bulb (Mombaerts et al., 1996). Using mice and rats as model organisms, it has been reported that any one OR is typically represented by two glomeruli (Mombaerts et al., 1996; Ressler, Sullivan & Buck, 1994; Vassar et al., 1994), which indicates that the number of glomeruli in the OB is approximately twice that of the number of *OR* genes in its genome. However, it is still unclear whether these findings can be extended to other mammals.

The glomerular layer of the OB can be classified into two domains, the dorsal (D) domain and the ventral (V) domain, based on the expression patterns of domain-specific marker genes (Imai & Sakano, 2007). The D domain is defined by the expression of the *OMACS* gene (Imai & Sakano, 2007; Oka et al., 2003), and the V domain is defined by the expression of the *OCAM* gene (Imai & Sakano, 2007; Yoshihara et al., 1997). All mammalian *OR* genes can be classified into two subfamilies, class I and class II, based on sequence similarities (Niimura & Nei, 2006). The OSNs expressing class I ORs are projected to the D domain of the OB, while OSNs expressing class II ORs are projected to both D and V domains (Imai & Sakano, 2007; Tsuboi et al., 2006).

Cetaceans are an order of mammals that originated in the early Eocene epoch and they derive from terrestrial artiodactyls (Thewissen et al., 2009). Extant cetaceans are classified into two monophyletic suborders, Odontoceti (toothed whales) and Mysticeti (baleen whales). Modern cetaceans are known to have reduced the olfactory capabilities profoundly during their evolution, and living odontocetes have no nervous system structures that mediate olfaction (Oelschläger, Ridgway & Knauth, 2010). On the other hand, at least some species of mysticetes have a fully equipped olfactory system and OB (Thewissen et al., 2011), but the number of functional *OR* genes is remarkably reduced. Terrestrial mammals, including cows, which are terrestrial relatives of whales, possess approximately 1,000 intact *OR* genes (Niimura, Matsui & Touhara, in press; Niimura & Nei, 2007). By contrast, minke and Antarctic minke whales (*Balaenoptera acutorostrata* and *B. bonaerensis*) possess only 60 intact *OR* genes (Kishida et al., 2015; Yim et al., 2014), and 56 of these are included in the class II OR subfamily (Kishida et al., 2015). In addition, genomic analyses have revealed that all modern mysticetes lack functional *OMACS* genes (Kishida et al., 2015). Based on these findings, it appears that, although mysticetes have fully equipped olfactory systems, their OB lacks the D domain (Kishida et al., 2015).

These molecular data suggest that mysticetes lack glomeruli on the dorsal side of their OB. In addition, because mysticetes possess a very small number of *OR* genes, it is expected that the number of glomeruli in their OB is also very small. However, no detailed study of the distribution and organization of glomeruli in mysticete OB has been reported to date. In this study, we provide the distribution of glomeruli in bowhead whales (*Balaena mysticetus*) and present data that test whether the mysticete OR:glomeruli ratio compares with the 1:2 ratio observed in mice and rats.

## Materials and Methods

Tissues of bowhead whales, details of which are shown in Table 1, were sampled from subsistence hunts in northern Alaska, USA, under NOAA/NMFS permit 814-1899. Whale OBs were fixed in 10% buffered formalin and processed using standard histological techniques. Section thickness was 6 µm. Details regarding laboratory procedures are described by Thewissen et al. (2011).

**Table 1:** Specimens studied.

Specimen no.	Species	Sex	Length (m)	Sampling date	Sectional plane	No. of stained sections
09B11	*Balaena mysticetus*	Female	7.2	Sep. 11, 2009	Coronal	5
09B14	*Balaena mysticetus*	Female	10.2	Sep. 14, 2009	Horizontal	1

Glomeruli are labeled by the expression of olfactory marker protein (OMP) (Danciger et al., 1989; Smith et al., 1991). The ImmunoCruz goat ABC staining system (catalog number sc-2023; Santa Cruz Biotechnology, Inc., Dallas, Texas, USA) and a rabbit polyclonal anti-OMP antibody (catalog number sc-67219; Santa Cruz Biotechnology, Inc., Dallas, Texas, USA) were used for immunohistochemistry, following the standard protocol attached to the ABC staining system kit. Antibody dilution was 1:150. The DAB-stained sections were counterstained with thionin, and then mounted on permanent slides. The number of glomeruli on each slide was counted manually, as shown in Figs. S1–S5. The numbers of glomeruli between these slides were estimated by the following formula:

\begin{eqnarray*} \displaystyle [f(n-m+1)+\mathit{rm}]/(n+1)&&\displaystyle \end{eqnarray*}

where, *n* is the number of estimated slides between counted slides (slide A and slide B), *f* is the number of glomeruli in slide A, *r* is the number of glomeruli in slide B, and the number of glomeruli on the *m*-th slide among *n* slides is estimated (*m* = 1, 2…, *n*).

In order to reconstruct a three-dimensional (3D) image of the OB, horizontal sections of the whole OB of a bowhead whale (specimen number 09B14) were prepared and every 5th slice was stained with thionin, mounted on permanent slides and photographed. Using AMIRA software (FEI Visualization Sciences Group, Burlington, Massachusetts, USA) ver. 5.4.1, these images were aligned with manual adjustments, and 3D reconstructed. A STL-formatted image of the 3D bowhead whale OB can be obtained under the following link (http://dx.doi.org/10.6084/m9.figshare.1295197).

We downloaded the bowhead whale genome assembly (Keane et al., 2015), and the *OR* genes were identified using TBLASTN program ver. 2.2.29 (Altschul et al., 1997). For details of *OR* gene identification and class I/II classification, we followed the methods used for identifying minke whale *OR* genes by Kishida et al. (2015).

## Results and Discussion

**Figure 1:**
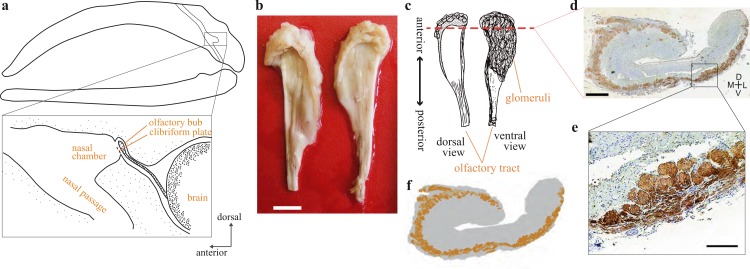
Olfactory bulb of the bowhead whale brain. (A) Diagram of the location of olfactory bulb in a sagittal section through the balaenid skull (modified after Thewissen et al. (2011)) (B) Dorsal view of the left and right OBs of bowhead whale (specimen 09B14). Scale bar, 10 mm. (C) Diagram of the dorsal and ventral view of the bowhead whale right OB. Coronal section (D) was cut at approximately the red dashed line. (D) Coronal section of the right olfactory bulb of bowhead whale specimen no. 09B11 (section195c). Glomeruli were stained with DAB using anti-OMP antibody, and the whole tissue was counterstained with thionin. D, dorsal; L, lateral; M, medial; V, ventral. Scale bar, 1 mm. (E) Details of glomeruli, enlarged the boxed region in (D). Scale bar, 240 µm. (F) A schematic view of the distribution of glomeruli of the coronal section of the bowhead whale OB.

Figure 1 shows OB glomeruli distribution patterns of bowhead whales. The shape of cetacean OB is not similar to that of terrestrial mammals, such as mice, in having a olfactory ventricle that is wide open dorsally, and with few glomeruli on the dorsal side of the OB. This finding is consistent with our genomic data showing that modern mysticetes lack receptors and marker proteins that are specific to the D domain of the OB (Kishida et al., 2015). We conclude that, from both genomic and morphological points of view, mysticete OB lacks the D domain. D domain-ablated mice fail to show innate avoidance behavior against odors of predators and spoiled foods (Kobayakawa et al., 2007), and it is possible that bowhead whales lack olfactory capabilities related to innate avoidance behaviors against such odors.

**Figure 2:**
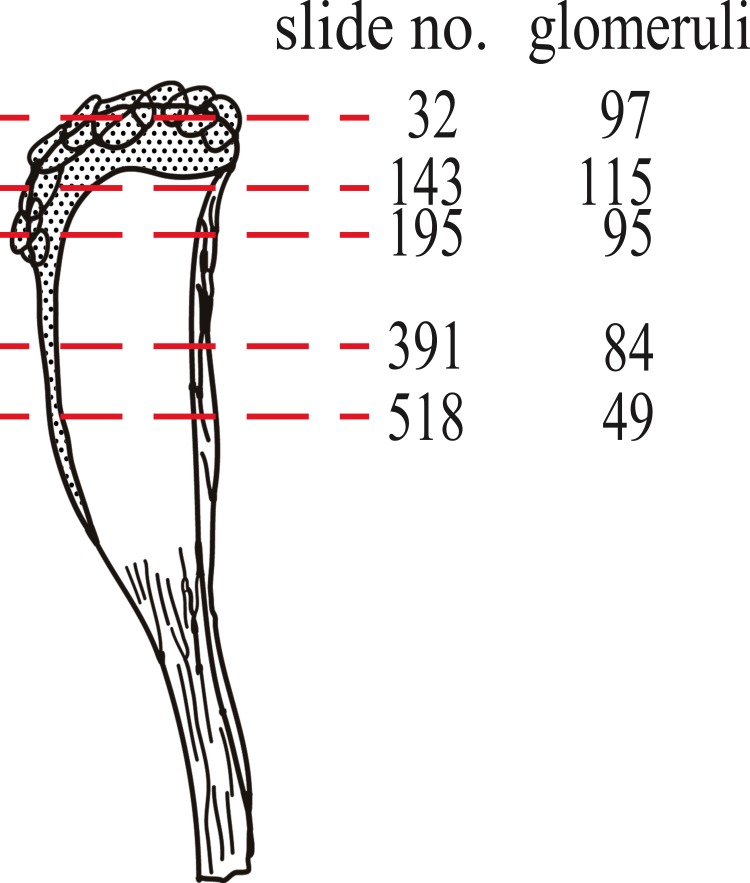
Nos. of glomeruli in five coronal sections investigated in this study. Sections were cut at approximately the red dashed lines. Detail pictures of the sections are available as Figs. S1 (slide no. 32), S2 (slide no. 143), S3 (slide no. 195), S4 (slide no. 391) and S5 (slide no. 518).

To test the OR: glomeruli ratio in mysticetes, we counted the number of glomeruli on five coronal sections, as shown in Fig. 2. We observed that the numbers of glomeruli shown in Fig. 2 is likely to be an underestimate of the actual number because some glomeruli cannot be discriminated clearly and were not counted. Generally, four coronal sections were mounted in one slide, and the thickness of each section was 6 µm. It is estimated that 10 slides, containing 40 sections, correspond to 240 µm. Because glomeruli are generally less than 240 µm in diameter (Fig. 1E, Figs. S1–S5 (coronal sections) and S6 (a horizontal section)), we expected that new glomeruli should appear at most every 10th slide. Therefore, we roughly estimated the number of glomeruli in approximately every 10th slide (Table S1). Surprisingly, this calculation for bowhead whale OB results in approximately 4,000 glomeruli, a number much higher than that of mice (1,600–1,800) (Royet et al., 1988; Taniguchi et al., 2003). We nonetheless consider this value to be an underestimate as explained above, and because the slides posterior of slide 518 were not examined (most of the glomeruli are located anterior of this slide).

Whole genome sequence data are required to obtain the repertoire of *OR* genes. Recently, a bowhead whale genome assembly was published (Keane et al., 2015) and we identified the *OR* gene repertoire in this genome assembly. Eighty intact and 11 truncated (i.e., lacking of 3′ and/or 5′ sequence(s) due to the fragmented scaffolds and/or contig gaps) *OR* gene sequences were identified (Table S2). Among these 91 *OR* sequences, only four genes were classified into class I (Table S2), including OR51E1 and OR51E2, which are not involved in olfaction (Kishida et al., 2015; Niimura, Matsui & Touhara, in press; Weng et al., 2006; Xu et al., 2000), supporting our view that bowhead whales OB lack the D domain. The number of *OR* genes in the bowhead whale genome is much fewer than the number of glomeruli in their OB, and thus we conclude that the OR: glomeruli ratio is not 1:2 in bowhead whales.

Humans are also reported to possess higher numbers of glomeruli (3,000–9,000) than the number of *OR* genes (350) (Maresh et al., 2008), similar to bowhead whales. Both humans and whales are known to have reduced their *OR* gene repertoires profoundly in their evolutionary pathways (Kishida et al., 2015; Matsui, Go & Niimura, 2010). It is possible that, in whales and humans, the evolutionary decline in glomerulus numbers proceeds at a slower rate than the decline of *OR* genes, which causes the aberrant ratio. Following this explanation, the ancestors of both whales and humans are expected to have a ratio of numbers of *OR* genes to glomeruli that is greater than 0.5. However, cows, a terrestrial relatives of whales for whom whole genome sequence data are available, possess approximately 1,000 *OR* genes (Niimura, 2009; Niimura & Nei, 2007), and other boreoeutherian mammals, including the last common ancestors of all modern boreoeutherians, also possess approximately 1,000 OR genes or less (Niimura, 2009). Therefore, we predict that whale ancestors would be expected to possess at most ∼1,000 *OR* genes, a much lower number than the number of glomeruli in whale OB. Similarly, the last common ancestors of all modern primates have been estimated to possess 585 *OR* genes (Matsui, Go & Niimura, 2010), a much lower number than the number of glomeruli in human OB. We speculate that the OR:glomeruli ratios are not fixed to 1:2 among mammals.

## Conclusion

Our results show that bowhead whale OB lacks glomeruli on the dorsal side, in accordance with molecular data showing that all modern mysticetes lack receptors and marker proteins specific to the D domain of the OB.

There is a much larger number of glomeruli in the bowhead whale OB than expected from the number of *OR* genes, indicating that the OR:glomeruli ratios are not always 1:2 among mammals.

## Supplemental Information

10.7717/peerj.897/supp-1 Figure S1 A coronal section of the OB of bowhead whale 09B11 (section 32). Glomeruli are labeled with anti-OMP antibody, and are indicated with arrows. Scale bar, 1,000 µm.

10.7717/peerj.897/supp-2 Figure S2 A coronal section of the OB of bowhead whale 09B11 (section 143). Glomeruli are labeled with anti-OMP antibody, and are indicated with arrows. Scale bar, 1,000 µm.

10.7717/peerj.897/supp-3 Figure S3 A coronal section of the OB of bowhead whale 09B11 (section 195). Glomeruli are labeled with anti-OMP antibody, and are indicated with arrows. Scale bar, 1,000 µm.

10.7717/peerj.897/supp-4 Figure S4 A coronal section of the OB of bowhead whale 09B11 (section 391). Glomeruli are labeled with anti-OMP antibody, and are indicated with arrows. Scale bar, 1,000 µm.

10.7717/peerj.897/supp-5 Figure S5 A coronal section of the OB of bowhead whale 09B11 (section 518). Glomeruli are labeled with anti-OMP antibody, and are indicated with arrows. Scale bar, 1,000 µm.

10.7717/peerj.897/supp-6 Figure S6 A horizontal section of the OB of bowhead whale 09B14 (section 134). Glomeruli are labeled with anti-OMP antibody. Scale bar, 1,000 µm. Left, anterior; right, posterior.

10.7717/peerj.897/supp-7 Table S1 Number of glomeruli on approx. every 10th slide. Nos. of glomeruli with slide nos. in parentheses are estimated by taking an average between the glomeruli-counted sections in front and in the rear.

10.7717/peerj.897/supp-8 Table S2 Loci of 80 intact and 11 truncated olfactory receptor genes on the *Balaena mysticetus* genome assembly (Keane et al., 2015)) identified in this study.

## Additional Information and Declarations

### Data Deposition

The following information was supplied regarding the deposition of related data: Figshare, http://dx.doi.org/10.6084/m9.figshare.1295197
