# Supplementary figures and images for: Organization and distribution of glomeruli in the bowhead whale olfactory bulb
Source: PeerJ. 2015 Apr 28;3:e897. doi: 10.7717/peerj.897 (PMC4419441; doi:10.7717/peerj.897)

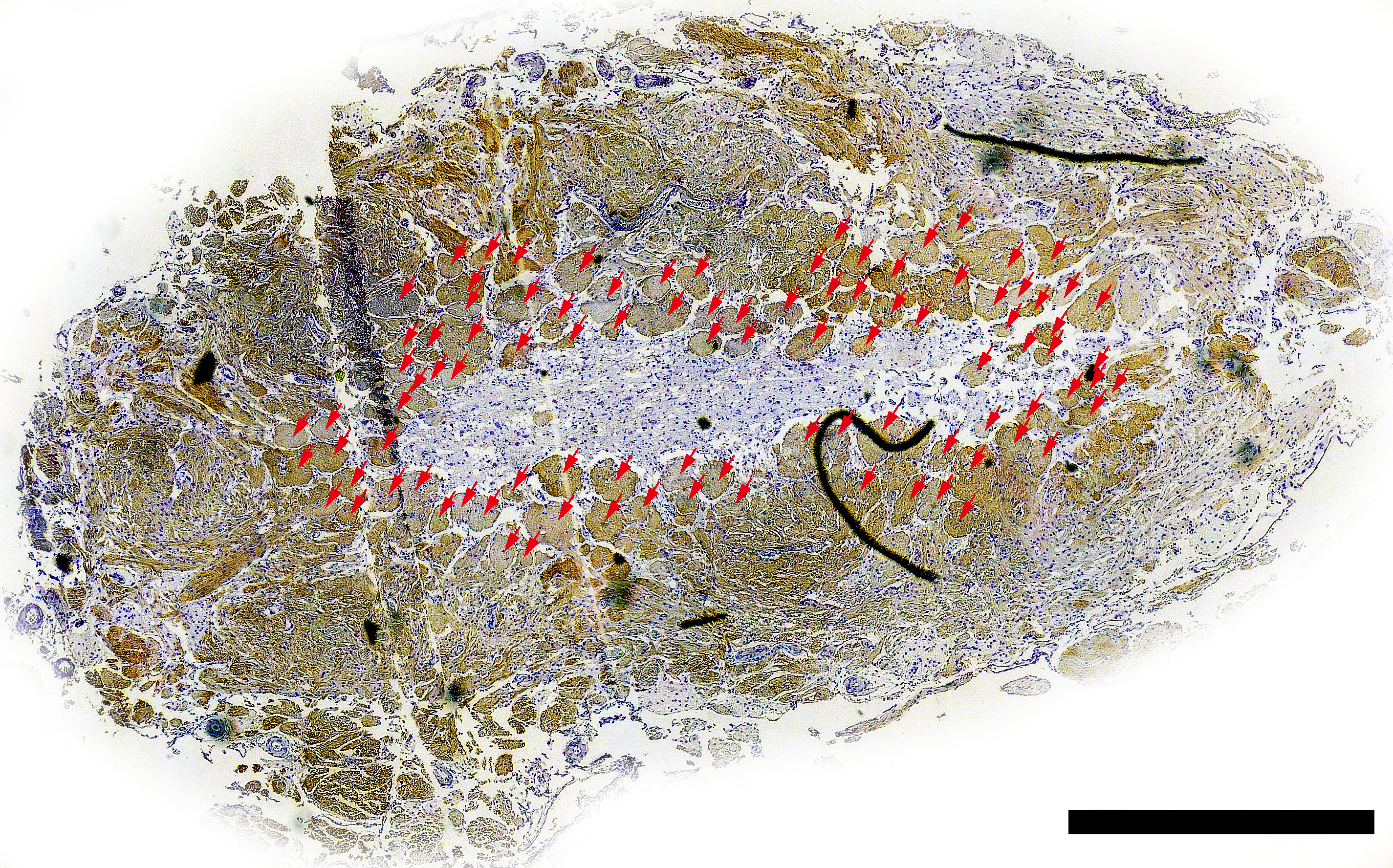

Supplement: Figure S1 [file peerj-03-897-s001.pdf]

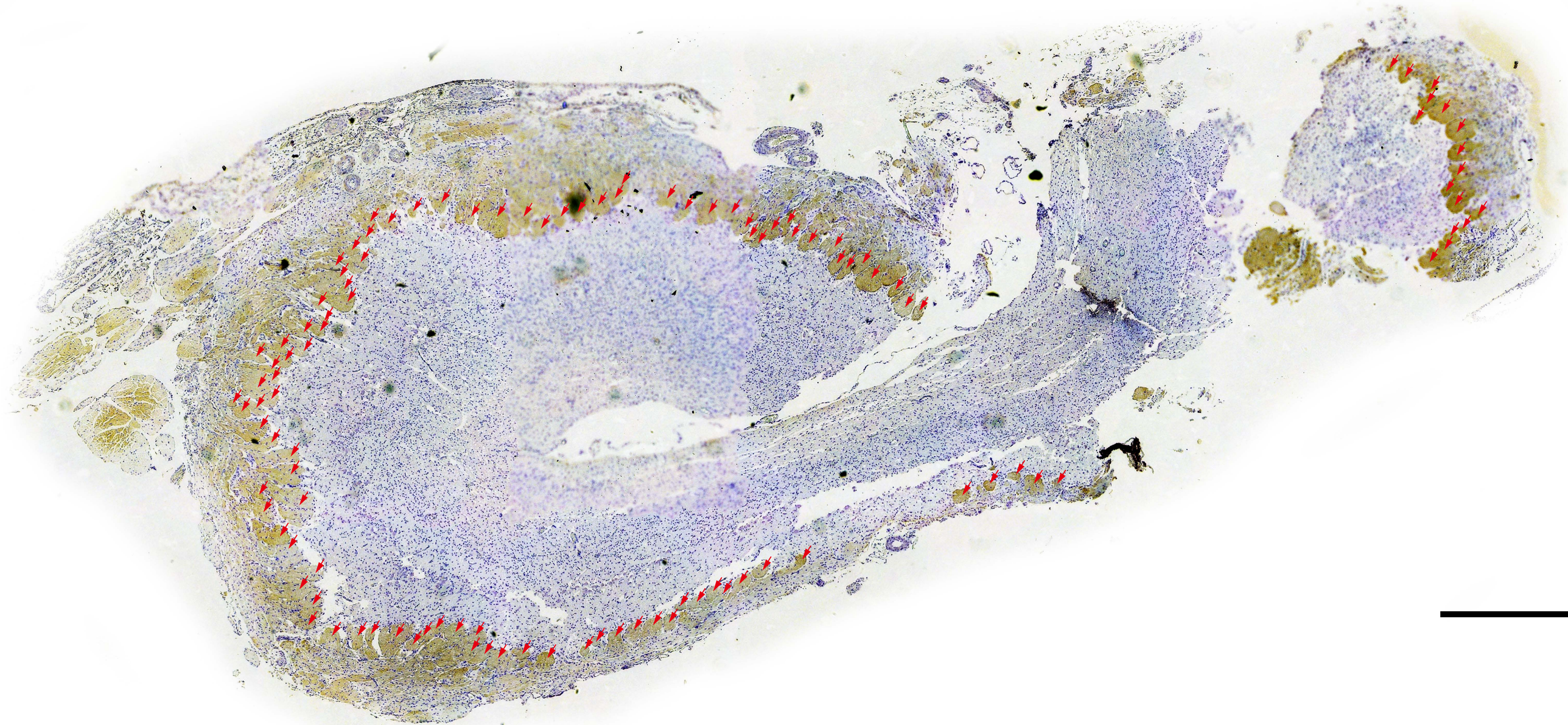

Supplement: Figure S2 [file peerj-03-897-s002.pdf]

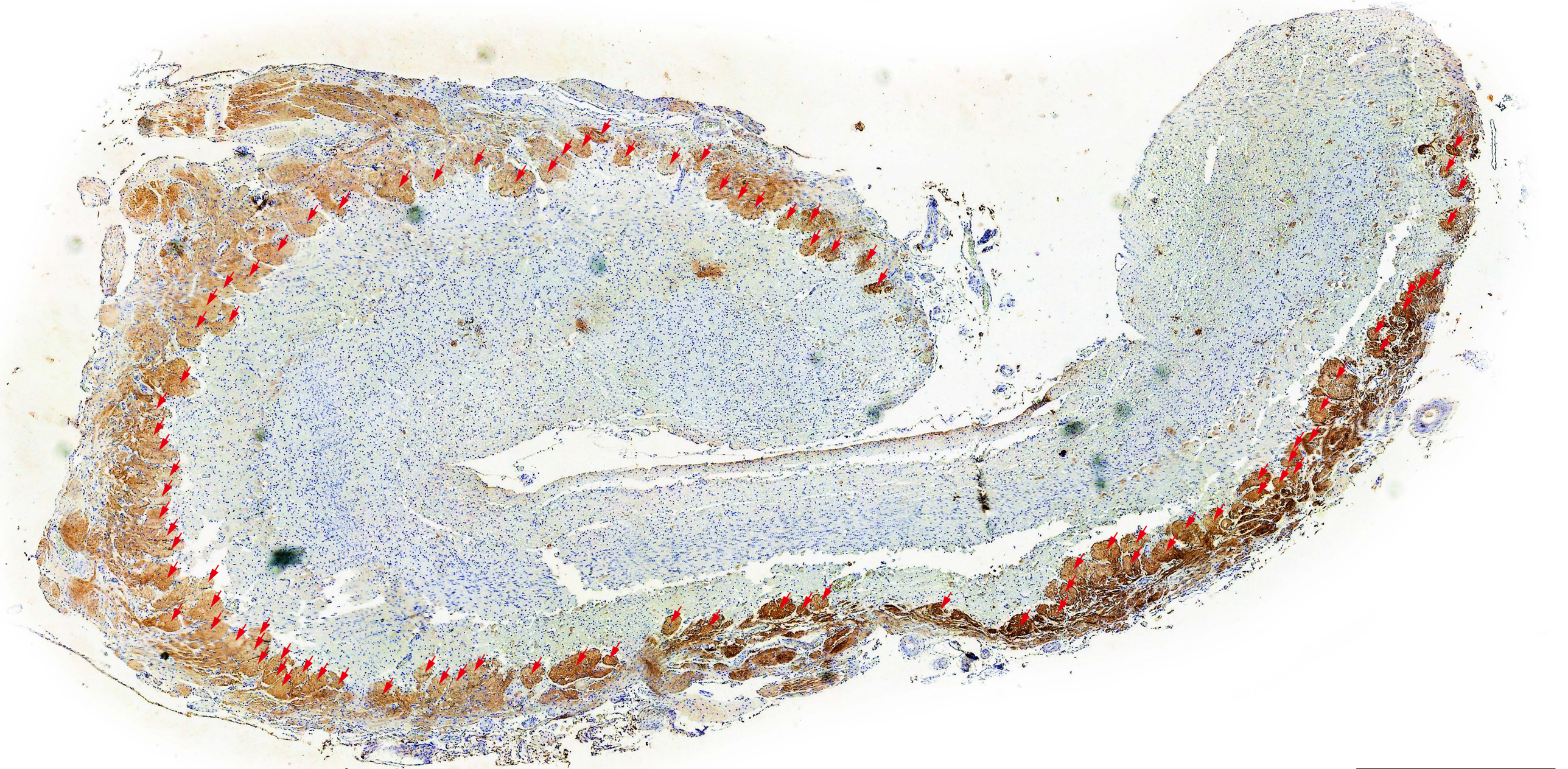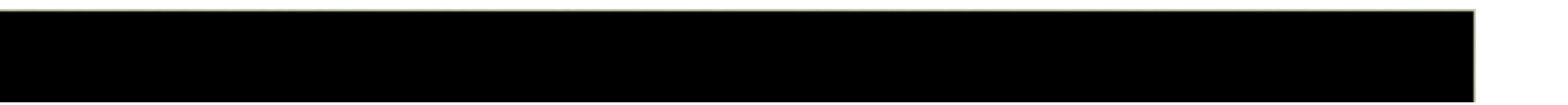

Supplement: Figure S3 [file peerj-03-897-s003.pdf]

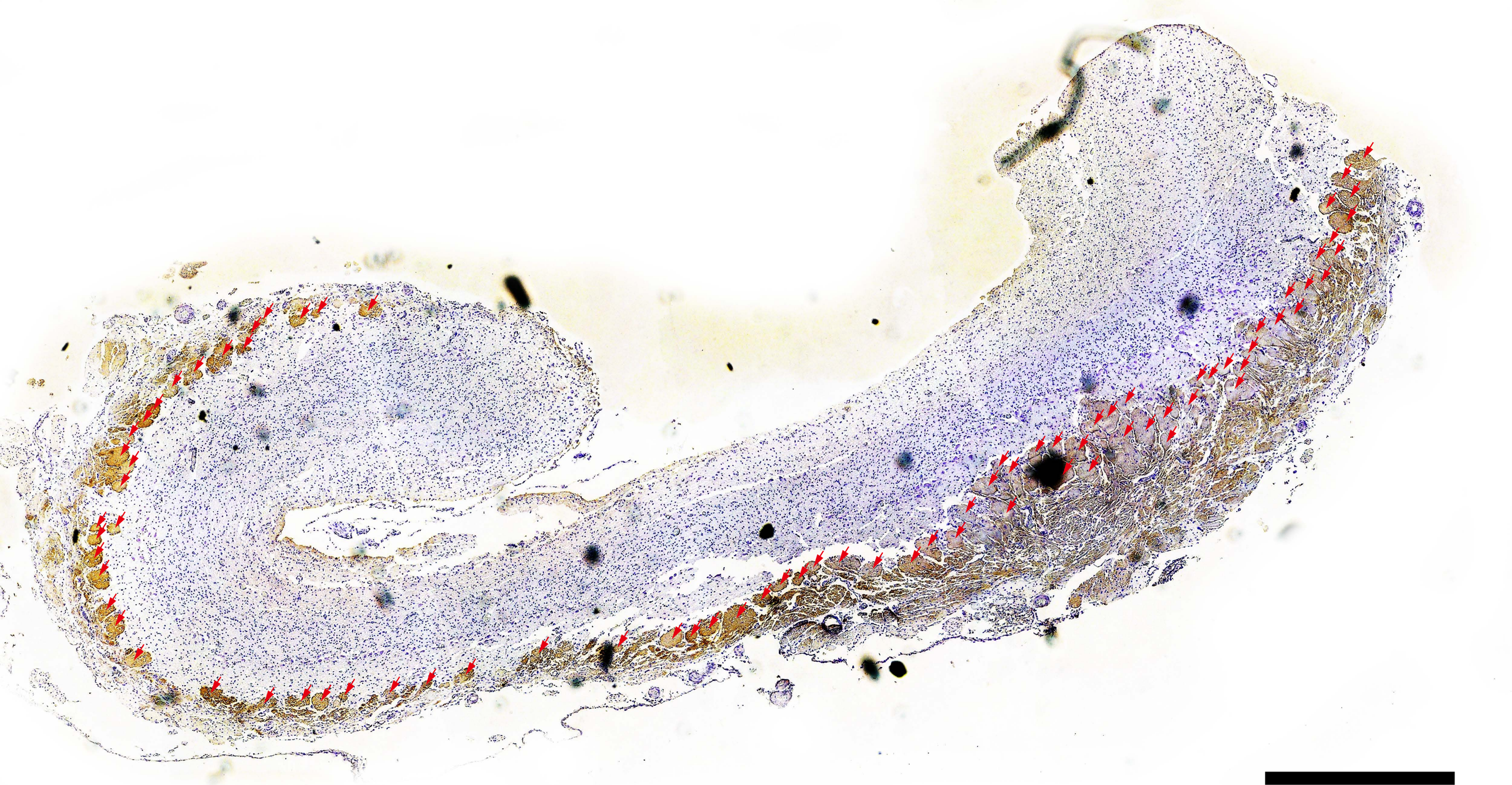

Supplement: Figure S4 [file peerj-03-897-s004.pdf]

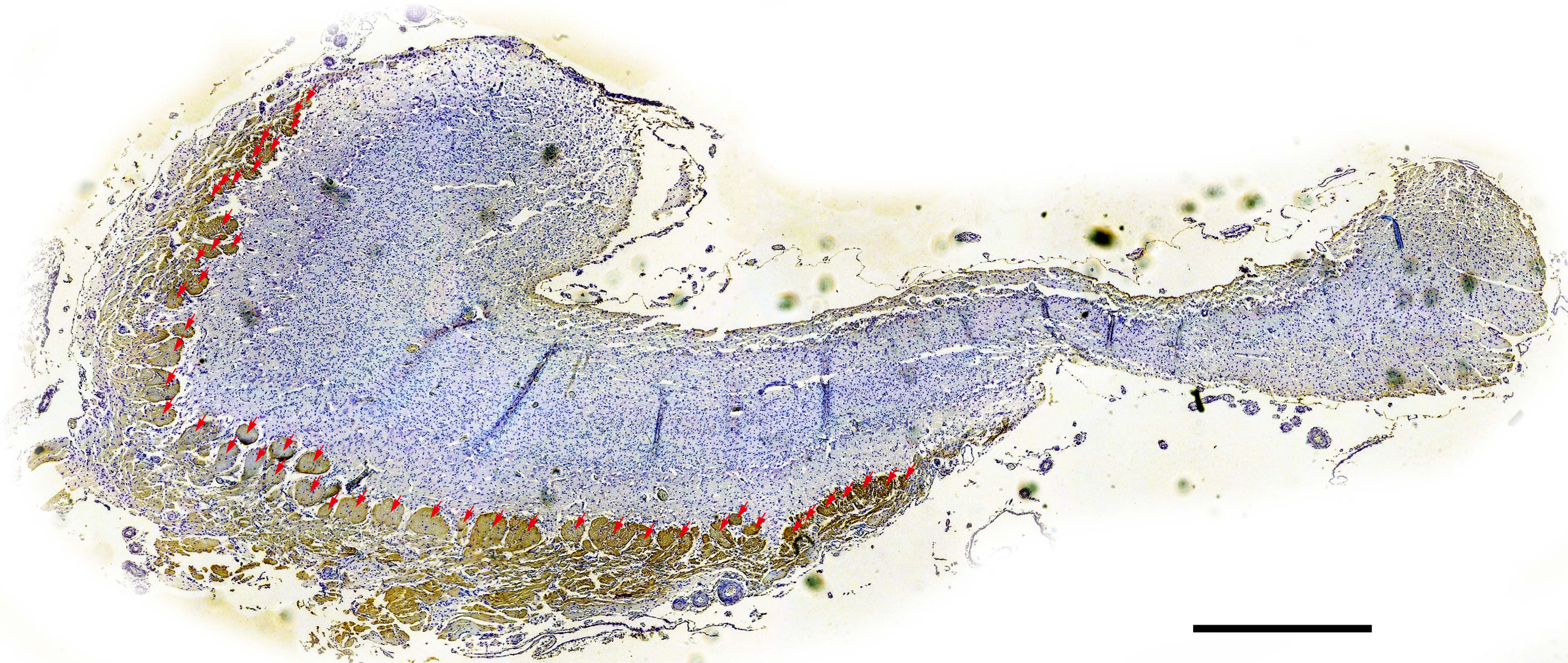

Supplement: Figure S5 [file peerj-03-897-s005.pdf]
